# Augmented Reality in Orthopedic Surgery Is Emerging from Proof of Concept Towards Clinical Studies: a Literature Review Explaining the Technology and Current State of the Art

**DOI:** 10.1007/s12178-021-09699-3

**Published:** 2021-02-05

**Authors:** Fabio A. Casari, Nassir Navab, Laura A. Hruby, Philipp Kriechling, Ricardo Nakamura, Romero Tori, Fátima de Lourdes dos Santos Nunes, Marcelo C. Queiroz, Philipp Fürnstahl, Mazda Farshad

**Affiliations:** 1 Department of Orthopedic Surgery, Balgrist University Hospital, University of Zurich, Zurich, Switzerland; 2 ROCS, Research in Orthopedic Computer Science, Balgrist Campus, University of Zurich, Forchstrasse 340, 8008 Zürich, Switzerland; 3 Computer Aided Medical Procedures (CAMP), Technische Universität München, Munich, Germany; 4 Computer Aided Medical Procedures (CAMP), Johns Hopkins University, Baltimore, MD USA; 5 Department of Orthopaedics and Trauma Surgery, Medical University of Vienna, Vienna, Austria; 6 Computer Engineering and Digital Systems Department, Escola Politécnica, Universidade de São Paulo, São Paulo, SP Brazil; 7 Laboratory of Computer Applications for Health Care, Escola de Artes e Humanidades, Universidade de São Paulo, São Paulo, SP Brazil; 8 Orthopedics and Traumatology Department, Faculty of Medical Sciences of Santa Casa de Sao Paulo, Sao Paulo, SP Brazil

**Keywords:** AR, Augmented reality, Computer-assisted surgery, HoloLens, VR, Hologram

## Abstract

**Purpose of Review:**

Augmented reality (AR) is becoming increasingly popular in modern-day medicine. Computer-driven tools are progressively integrated into clinical and surgical procedures. The purpose of this review was to provide a comprehensive overview of the current technology and its challenges based on recent literature mainly focusing on clinical, cadaver, and innovative sawbone studies in the field of orthopedic surgery. The most relevant literature was selected according to clinical and innovational relevance and is summarized.

**Recent Findings:**

Augmented reality applications in orthopedic surgery are increasingly reported. In this review, we summarize basic principles of AR including data preparation, visualization, and registration/tracking and present recently published clinical applications in the area of spine, osteotomies, arthroplasty, trauma, and orthopedic oncology. Higher accuracy in surgical execution, reduction of radiation exposure, and decreased surgery time are major findings presented in the literature.

**Summary:**

In light of the tremendous progress of technological developments in modern-day medicine and emerging numbers of research groups working on the implementation of AR in routine clinical procedures, we expect the AR technology soon to be implemented as standard devices in orthopedic surgery.

## Introduction

Technologic advances significantly influenced the way orthopedic surgery is practiced today and paved the way for recent innovations in surgical procedures. Complex surgical interventions, as needed for the treatment of musculoskeletal tumors, fracture reduction, corrective osteotomy procedures, or instrumentation adjacent to critical anatomic structures require precise preoperative planning and accurate intra-operative execution. Computer-driven approaches have been established in many areas of orthopedic surgery to support the surgeon in facilitating preoperative planning or improving surgical execution [1, 2]. Different technological innovations have been implemented in orthopedic surgery such as robotic surgery [3], 3D-printed patient-specific instrumentation (PSI) [1], navigation tools with tracking visualized on monitors [4], and the emerging technology of augmented reality which allow a human-computer interface [5]. Robotic surgery still has significant limitations that prevent broader clinical use, as devices are very expensive [6], spacious, and their setup and maintenance are time-consuming and cost-intensive [7]. PSI has proven as feasible tool with excellent postoperative results [8, 9]. However, the design and manufacturing of patient-specific instruments need up to weeks of lead time. Another drawback is that accurate navigated surgical execution requires extensive bone exposition [10].

AR is defined as “a system”, where the real world is enhanced with virtual computer-generated sensory impressions that appear to coexist in the same space as the real world [11]. These virtual impressions can be visual stimuli (e.g., holograms), and any other sensorial information such as sound. The AR technology originated from the military sector [11, 12] has been widely used commercially in the area of entertainment and gaming [13] and is now promoted for use in orthopedic surgery [14]. Recently, the FDA (U.S. Food and Drug Administration) approved the first AR applications for elective spinal surgery [15, 16].

Orthopedic surgical procedures require a vast amount of numeric and geometric information, such as angles of deformity [17], anatomic relations for instrumentation [18], vital parameters such as blood pressure for blood loss control [19, 20] or trajectory orientation for instrumentation and implant placement [21••]. In daily clinical practice, these parameters are analyzed preoperatively by the surgeon and made available via prints and/or digital imaging data on screens in the operating room. Still, information is lost between preoperative planning and the execution of the procedure.

AR represents a valuable solution to improve information transfer and consideration during surgery. Current AR approaches are mainly visual and use monitors [22] or head-mounted displays (HMD) [23•].

Recent publications on the topic evaluate the potential benefits of this technology, especially in regard to radiation exposure of the patient and staff, procedure time in the operating room, and improvement of accuracy of surgical execution. This review provides a comprehensive overview of the current state of the technology and recent research of AR in the field of orthopedic surgery (Table 1). The aim of the article is to elucidate basic principles and current applications of AR to orthopedic surgeons, who lack profound knowledge in computer sciences.

**Table 1:** Studies presenting AR applications in orthopedic surgery categorized in the areas spine, osteotomy, arthroplasty, trauma, and oncology

Author, Year	Category	Application	Registration Technology	Visualization	Description/results	Study model
Elmi-Terander et al. 2020[24••]	Spine	Pedicle screw navigation	Intraoperative cameraaugmented cone beam CT was recorded and tracked using skin markers	Monitor	20 patient with Augmented reality surgical navigation (ARSN) were matched with 20 freehand (FH) performed cases. Pathologies included scoliosis, kyphosis and other. A total of 262 ARSN and 288 FH screws were assessed. Correct screw position graded by Gertzbein scale was significantly higher in the ARSN 93.9% vs FH 89.6% group. Screws without a cortical bone breach were double in the ARSN 63.4% group compared to the FH 30.6%.	clinical study
Müller et al. 2020[25]	Spine	Pedicle screw navigation (L1 - L5)	Optic radiopaque markers attached to cadavers and included into preoperative plan.	HMD, Microsoft HoloLens	Accuracy evaluation of AR navigated screws in 3 lumbar spine cadavers showed no significant difference between entry points to pose tracking navigated instrumentation. 3.4±1.6 mm compared with 3.2±2.0 mm, and angular errors between trajectories 3D angle; p=.30 of 4.3°±2.3° compared to 3.5°±1.4°.	cadaver phantom model
Elmi-Terander et al. 2019 [22]	Spine	Pedicle screw navigation (Th1 - S1)	Intraoperative cameraaugmented cone beam CT was recorded and tracked using skin markers	Monitor	One orthopedic spine surgeon placed 253 lumbosacral and thoracic ARSN navigated pedicle screws in 20 patients. 15 (5.9%) patients had 2 - 4 mm breach (Gretzbein 2) misplacement, 2 medial and 13 lateral. Of those 13 screws, the diameter exceeded the pedicle.	clinical study
Liebmann et al. 2019[21••]	Spine	Pedicle screw navigation (L1 - L5)	Surface point cloud	HMD, Microsoft HoloLens	The mean errors of preoperative planning and navigated screw insertion were 3.38∘±1.73 for the screw trajectory orientation and 2.77±1.46 mm for the entry points. The mean time required for surface digitization was 125±27 s.	sawbone
Wanivenhaus et al. 2019[23•]	Spine	Spinal rod bending	Marker detection of screw head position	HMD, Microsoft HoloLens	AR navigation reduced total time spent on bending and inserting the rod compared to conventional bending by 20% (374±79 vs. 465±121 s, p=.01195). The navigation required less re-bending maneuvers (7/18 vs. 10/18 p>.05)	sawbone
Molina et al. 2019[15•]	Spine	Pedicle screw navigation (Th6 - L5)	Reflective patient marker attached to the spinous process.	HMD, Xvision Augmedics	Screw placement accuracy achieved with the AR system was 96.7% based on the Heary-Gertzbein scale and 94.6% based on the Gertzbein scale. Accuracy was non-inferior compared to manual computer-navigated pedicle insertion and robotics-assisted computer-navigated insertion. The AR system was superior to results obtained with freehand insertion.	cadaver study
Yoon et al. 2017[26]	Spine	Pedicle screw navigation (C2 - S2)	O-Arm (Medtronic Stealth S7, Medtronic Inc.)	HMD, Google Glass	Forty AR-navigated pedicle screws were placed in 10 patients. The average screw placement time was slightly shorter when the HMD was used (4.13 min with vs. 4.86 min without). The post‐procedure survey demonstrated that 79% of surgeon’s responses were positive. No complications occurred.	clinical study
Elmi-Terander et al. 2016[27]	Spine	thoracic pedicle screw navigation	Intraoperative cameraaugmented cone beam CT was recorded and tracked using skin markers	Monitor	Two neurosurgeons instrumented 94 ARSN pedicle screws on one side and freehand (FH) on the other of four cadavers. The study showed superiority of the ARSN when rated by Gertzbein grading ARSN 85% vs. FH 64%, P < 0.05.	cadaver study
Wu et al. 2014[28]	Spine	Entry point guidance for vertebroplasty	ARCASS system with marker tracking	camera-projector system	Navigation was superimposed onto the situs using an entertainment projector. The application was verified on a dummy and later animal cadaver. After final adjustments three patients underwent spinal surgery. The group reported reduced time of finding the entrance point and reduced radiation. The average error of the targeted entry point was 9.1 mm. Body movement impacts the registration.	animal cadavers (pig), clinical case report
Kosterhorn et al. 2017[29]	Osteotomy	Navigation for pedicle subtraction osteotomies	markers attached to spinous process of L1	head-up display implemented into surgical microscope	The surgery was successfully executed on a 56-year-old female patient with severe painful scoliotic deformity. No complications or neurologic deficits were observed. Pain was reduced by up to 75%.	clinical case report
Fallavolita et al. 2016[30]	Osteotomy	Intraoperative assessment of mechanical axis	camera-augmented c-arm with biplanar marker beneath limb	Monitor	The method could visualize the mechanical leg axis with strong correlation compared to ground-truth CT data according to Pearson's correlation coefficient R of 0.979. Surgeons required three x-rays to visualize the mechanical axis reliably.	cadaver study
Ogawa et al. 2020 [31••]	Arthroplasty (hip)	Navigation for acetabular cup placement	markers attached to bone and instruments	Monitor	46 patients were treated with total hip replacement using AR- navigation for acetabular cup placement. Radiographic evaluation showed a smaller plan to outcome error for inclination in in the AR group (x-ray; 2.3° ± 1.4° versus 3.9° ± 2.4°, respectively; p = 0.009 and CT; 1.9° ± 1.3° versus 3.4° ± 2.6°; p = 0.02). There was no difference in anteversion between the groups.	clinical study
Alexander et al. 2020[32]	Arthroplasty (hip)	Navigation for acetabular cup placement	camera-augmented c-arm	Monitor	AR technique was significantly more accurate for achieving target inclination p=0.01 and anteversion p=0.02 compared to fluoroscopic instrumentation. The AR method was faster 1.8 ± 0.25 vs 3.9 ± 1.6 minute; p < .01.	sawbone
Tsukada et al. 2019[33]	Arthroplasty (knee)	Navigation for tibial bone resection	markers attached to bone and instruments	Monitor	The results presented comparable accuracy of this AR system for bone resection compared to conventional navigation systems (not specified). The study tested a proof of concept and is not recommended for clinical use at this state of the system by the authors.	sawbone
Ogawa et al. 2018[34]	Arthroplasty (hip)	Navigation for acetabular cup placement	markers attached to bone and instruments	Monitor	AR navigated acetabular cups were more accurate in terms of planned anteversion compared to using a conventional goniometer. AR 2.7° vs goniometer 6.8°; p < .0001)	clinical study
Fotouhi et al. 2018[35]	Arthroplasty (hip)	Navigation for acetabular cup placement	camera-augmented c-arm	Monitor	The group introduced a new innovation for acetabular cup placement. Planning requires only 2 stereo x-rays. Evaluation showed minimal error for translation 1.98 mm, anteversion 1.10°, and abduction 0.53 °.	sawbone
Liu et al. 2018[36]	Arthroplasty (hip)	Drilling of guide hole into femur for hip resurfacing	marker based tracking and point-cloud by depth camera	HMD, Microsoft HoloLens	AR navigation showed no significant difference of entrance point error between different executingstudy participants. Drilling trajectory error was insignificant p=0.9.	sawbone
Cho et al. 2018[37]	Oncology	AR vs. Conventional navigation for visualization for bone tumor resection	Optical Marker + Tablet camera	Monitor / Tablet	On 36 porcine pelvic cadavers bone tumor resection was performed using AR navigation and conventional resection. The resection error in the AR group was 1.59 ± 4.13 mm with no tumor violation. In the conventional group the error was 4.55 ± 9.7 mm with 14% error > 9mm or tumor violation.	animal cadavers (pig)
Cho et al. 2017[38]	Oncology	AR navigation vs. conventional method without navigation for visualization and resection of bone tumors	optical surface marker	Monitor / Tablet, Microsoft Surface 3 pro	Comparison between conventional and AR method for 164 bone tumor resections in 82 pig femurs. The target resection margin was 10 mm. For the AR group the mean error was 1.71 mm (90% < 10 mm) with no tumor violation, for the conventional group 2.64 mm (70% < 10 mm) with three tumor violations. After method validation the group performed one clinical case with a low-grade osteosarcoma in the diaphysis of the tibia. With AR - navigation, margins were 1.4 cm and 1.7 cm.	animal cadavers (pig), clinical case report
Choi et al. 2017[39]	Oncology	AR navigation vs. freehand visualization and resection of bone tumors	markers attached to bone and instruments	Monitor / Tablet, Microsoft Surface 3 pro	60 porcine pelvises were tumor resected using AR-navigation and a free-hand method. The conventional group achieved an accuracy of 7.11 ± 4.30 mm with 25% tumor violation vs. the AR group 9.85 ± 1.02 mm with no tumor violation	animal cadavers (pig)
Fritz et al. 2013[40]	Oncology	AR visualization for biopsy intervention	not specified	Monitor	MR imaging was visualized and showed the tumor. The method achieved successful osseous biopsy in 16 of the 16 target lesions (8 pelvis, 8 thoracolumbar spines). One attempt was sufficient for accurate targeting of all lesions (lesion size 2.2 cm, 1.1-3.5 cm). Time required for biopsy was 38 minutes (20–55 minutes)	cadaver study
Weidert et al. 2019[41]	Trauma	Distal interlocking during intramedullary nailing	camera-augmented c-arm, optical marker	Monitor	3 surgeons with different experience levels performed distal interlocking with 7 pairs of cadaveric bovine forelimb bones. No difference was detected in procedure time p=0.96 and drilling quality p=0.12. Radiation exposure was significantly reduced using the AR application by less experienced surgeons p<0.05 .	animal cadaver (bovine)
von Heide et al. 2018[42]	Trauma	Fluoroscopic visualization during different types of osteosynthesis surgery	camera-augmented c-arm, optical marker	Monitor	The study compared traditional c-arm with the camera-augmented c-arm superimposing x-ray imaging on a monitor in 45 and 28 trauma surgeries. The radiation exposure was reduced by 46% with the AR-method. The surgical time was not significantly altered.	clinical study
Shen et al. 2013[43]	Trauma	Hemi-Pelvic plate bending with AR navigation	Optical marker	Monitor or HMD, nVisor MH60, NVIS Inc., VA	For 6 patients for unilateral pelvic fractures a preoperative computer assisted reduction plan with patient specific plate bending was conceived. With AR navigation plates were bent preoperatively and used intraoperatively. A significant reduction in surgical time of 10 minutes was noted and fractures were reduced successfully. No complications occurred.	clinical study
Ortega et al. 2008[44]	Trauma	Fluoroscopic visualization during ORIF and pedicle screw placement	c-arm with connection to HMD	HMD, MicroOpitcal	50 patients at multiple centers were treated for different orthopedic procedures visualizing intraoperative fluoroscopy on a HMD. The view left the operative field 207 in conventional visualization and 5 times with HMD visualization.	clinical study

## Basic Principles of AR

### Data Preparation for Visualization of Radiologic Imaging and Navigation

In case of surgical navigation, the relevant information for the surgeon is typically extracted preoperatively from two-dimensional (2D) or three-dimensional (3D) radiologic imaging. The virtual model then consists of a computed 3D anatomy model including data about the surgical navigation to execute critical surgical steps. Fürnstahl et al. [45••] described the process of data preparation and surgical planning for the purpose of 3D surgical navigation of long bone deformities as follows.

A CT scan of the pathologic bones is acquired in high-resolution with a slice thickness of 1 mm or smaller. The data is then imported in a commercial image processing software such as Mimics (Mimics Medical, Version 19; Materialise, Leuven, Belgium). The anatomical region of interest is segmented from the soft tissue using density-based thresholding and region-growing functions. A 3D triangular surface model is generated from the segmented image using the Marching Cube algorithm [46]. The 3D model is then imported into a surgical planning software in order to elaborate a computer-assisted surgical plan by stepwise simulation of the surgery. Machine-learning approaches have shown good results for improving automatization of this process [47–52], but the gold standard for clinical-grad surgical planning remains human-based planning performed by experts [53••].

### Registration and Tracking

Registration is the process in which the visualized computer-generated object, which can be a radiologic image or a modality of navigation, is superimposed and oriented into situs in the correct position. After registration, tracking enables the visualized object to stay in the right position when moving and to adapt to the user position as well as to the detect instruments and their orientation and movement in three-dimensional space. Tracking requires the AR-system to reference the visualization or instrument from its original registration in a spatial room. For instruments, this is referred to as pose reconstruction. Low accuracy of registration and motion tracking is one of the main pitfalls of this technology [21••] for surgical use. The methods for registration presented in this review are camera-augmented c-arm registration [22, 44, 54–56], marker-based registration [25, 28, 34, 38], and surface registration [21, 23]. Navab et al. [54••] first engineered the camera-augmented surgical c-arm and discovered its potential for augmented reality in the operating room. This system registers intraoperatively acquired X-rays to the camera image of the operation field in a 3D coordinate system shared with the AR-head-mounted-display (HMD), such that the X-rays can then be visualized in real-time [56].

Marker-based registration is based on the registration of marker positions in a 3D coordinate system in relation to the computed reality augmentation, usually a 3D model of the anatomical region of interest. Its accuracy depends on exact positioning of the markers [25, 34, 38, 57].

Liebmann et al. [21••] first introduced intraoperative radiation-free registration by surface digitalization using only an AR-HMD and a pointing device to navigate pedicle screw instrumentation. Their approach registers a CT-based 3D preoperative plan by superimposing the 3D model to the intraoperative bony surface. A marker senses the surface of the exposed bone surface and samples a point cloud intraoperatively. When sufficient information is collected, the unique surface pattern is recognized, and the 3D model including the surgical plan is superimposed on the surgical field using iterative closest point registration [58].

### Visualization

Within the field of human-computer interaction, augmented reality can be understood as a class of displays [59]. The fusion between artificial information and real-world images is performed by either optical or video see-through techniques [11]. There are three main approaches to implementing those displays: head-mounted displays (HMD), monitors, and projectors [11]. Display weight, size, and resolution favor the use of an HMD as a simple method of visualization. HMD with a see-through display visualizes information into the field of view of the user [60].

Regarding visualization, there are two main approaches. First, the visualization can be independent from any spatial relation. This application is mainly useful for displaying additional information such as numeric data [61]. A more sophisticated approach displays a visualization depending on associated spatial positions. Thus, the image changes according to the position of the user. This requires position tracking of the HMD. If the device itself is capable to determine its spatial position by means of integrated sensors, it uses inside-out tracking [62]. On the contrary, outside-in tracking uses an external camera system to detect and track the position of the AR device. In outside-in tracking additional hardware is required, and occlusions may occur, which are two disadvantages of this system [63].

Most commercial AR-devices have originally been developed in the area of entertainment and gaming [13]. Therefore, the commercially available hardware itself is hardly appropriate for clinical use since its original capacity was not intended for high-accuracy visualization. The accuracy is mainly dependent on two factors: registration and exact spatial location for tracking. Liebmann et al. [64•] has pointed out the problem with low-fidelity tracking for navigation purposes resulting in drifting virtual models in orthopedic surgery. In 2019, an HMD device engineered for surgical use, Xvision (Augmedics, Arlington Heights IL, USA), was the first to be cleared by the FDA for spinal surgery navigation [15•] and since early 2020, the Microsoft HoloLens has been approved to be used for spinal surgery navigation in a Swiss first-in-man clinical study by Swissmedic.

## Clinical Applications of AR

### Spine Surgery

The spinal cord as well as its emerging spinal nerves and accompanying vessels are prone to iatrogenic injury during instrumentation due to the close proximity to the bony structure of the spine. Mispositioning of pedicle screws during spinal fusion surgery can result in neurological or vascular injury with severe long-term sequelae [22]. Therefore, the majority of AR applications in spine surgery address the surgical navigation of pedicle screw instrumentation [15, 16, 21, 22, 25, 26, 28].

Yoon et al. [26] placed forty pedicle screws using the Google Glass (Foxconn, Google, Mountain View, CA, USA) as a head-mounted display (HMD) in 10 consecutive patients. The study group instrumented cervical, thoracic, and lumbar pedicle screws navigated with the Medtronic Stealth S7 (Medtronic Inc., Littleton, Massachusetts) image-guidance system with radiologic imaging using the O-ARM (Medtronic Inc) and visualized with the HMD. The HMD had a voice control feature to control the information to be displayed. The registration, tracking, and navigation were performed by the Medtronic Stealth S7 system. This feasibility study described the use of HMDs during the procedure of pedicle screw instrumentation as safe. No complications were reported in the results.

Molina et al. [15•] placed Th6 to L5 pedicle screws in five male cadaver torsos using the Xvision (Augmedics, Arlington Heights IL, USA). The group navigated 120 pedicle screws and graded accuracy using the Gertzbein scale (GS) [65], a combination of that scale and the Heary classification [66], referred to in this paper as the Heary-Gertzbein scale (HGS). Overall accuracy when using the AR system was 96.7% based on the HGS and 94.6% based on the GS, which is similar to the accuracy reported for computer-navigated pedicle instrumentation. User experience evaluated with the user experience questionnaire [67] was rated as excellent in terms of usability.

Elmi-Terander et al. [22] navigated 253 pedicle screws from Th1-S1 in a clinical study of twenty patients. They used a modified version of the camera-augmented c-arm [55] and also graded accuracy using the GS and achieved overall accuracy of 94.1%. No screws were assessed as Gertzbein grade 3. The group described a decreased time for instrumentation once experience was gained. From a starting time of 17 min required for screw placement, the average instrumentation time dropped to 1.8  ±  0.9  min with increased experience with the navigation system.

The same research group performed a follow-up study in the form of a case-control-study [27] consisting of 20 AR-guided versus 20 free-hand instrumented pedicle screws, which confirmed most of their preliminary findings. The clinical accuracy of AR navigation was 93.9% and thus slightly higher compared to the free-hand group with 89.6%. The percentage of perforation was only half as high with AR compared to free-hand screws. No significant difference in instrumentation time could be shown between the groups.

Liebmann et al. [68] developed a new registration method to superimpose a 3D model of the patient vertebra together with planning information including pedicle screw insertion point and trajectory. Their idea was to register a point cloud of the exposed bone surface using a marker-tracked pointing device to the 3D model of the surgery plan. The group navigated L1–L5 screws using the HoloLens on spine sawbone models and reported an accuracy of 3.38°± 1.73 for the screw trajectory orientation and 2.77 ± 1.46 mm for the entry point localization. The mean time required for surface digitization was 125 ± 27 s.

Müller et al. [25] described an image-based registration approach, which was evaluated in a cadaver study. Three spine cadavers were embedded in opaque agar gel to simulate a lumbar torso. They attached optical markers with radiopaque parts to specified anatomical locations on the cadaver and acquired a CT scan with a cone beam CT device. After segmentation and registration of the bony anatomy, the transformation between markers and anatomy enabled real-time overlay of the surgery plan for pedicle screw instrumentation. The proposed approach could achieve an accuracy in pedicle screw placement comparable to navigation with high-precision optical systems.

Wu et al. [28] superimposed radiologic imaging onto the patient’s skin using a commercially available entertainment projector. The used system visualized the patient’s anatomy to guide needle instrumentation for vertebroplasty. They evaluated the system on a synthetic phantom and verified precision of the system on an animal cadaver. Later, he assessed accuracy of inserting points during vertebroplasty in three clinical trial participants. The mean transition error in entry point location was 4.4 mm, and the system reduced the time of finding the entry point by 70%. They, however, noted that adipose tissue during surgery influences the accuracy of this system since the overlying skin, where the markers are attached, is mobile.

### Osteotomies

Precise surgical execution of osteotomies is crucial in corrective procedures to reconstruct the physiologic anatomy [30]. Particularly, complex corrective osteotomies consisting of multiple oblique or curved osteotomy planes are challenging to perform without support through surgical navigation [29, 45, 69, 70].

Fallavolita et al. [30] presented a method to visualize the mechanical leg axis intraoperatively using AR. The group used the camera-augmented c-arm [55] to create a panorama view of the hip center, knee, and upper ankle joint based on three X-ray images. Twenty-five cadaver legs with random varus or valgus deformities were used to validate their method and confirmed it to ground truth CT data with no statistically significant difference. The group stated that the method allowed reliable tracking of the leg axis intraoperatively requiring only 3 X-rays.

Kosterhorn et al. [29] presented a case report about an AR application integrated into a surgical microscope through an HMD. Surgical planning was visualized in situ and allowed the study group to navigate the osteotomy planes of pedicle subtraction osteotomies. The anatomy was registered to intraoperative accessible landmarks of the vertebral body. The procedure itself is a high-risk intervention consisting of invasive osseous reduction of the vertebral body in proximity to the neuronal structures of the spine. The method was first simulated on a sawbone spine model and later implemented in the operating room. In the presented case, the surgeons resected a 27° posterior wedge of the Th1 vertebral body and reported good match with the virtually overlaid navigation template. The pathologic segmental kyphosis Th11–12 improved from 45 to 5°. No complications or neurologic deficits were observed.

### Arthroplasty

In arthroplasty, exact implantation of the prosthetic components with respect to the patient’s anatomy is a main contributor to successful outcomes, functional recovery, and longevity [32, 34, 36]. Three studies performed on sawbones evaluated AR navigation of arthroplasties as more accurate than free-hand procedures in hip [32, 36] and knee surgery [33].

Ogawa et al. [34] performed 56 total hip arthroplasties in 54 patients superimposing cup orientation through a smartphone into the surgical filed (group 1) and using a goniometer (group 2) for navigating placement and orientation of the acetabular cup component. Three months after implantation, a CT scan was acquired for assessment of the surgical accuracy. AR navigation was significantly more accurate in terms of radiographic anteversion compared to the goniometer method (2.7° vs. 6.8°). The AR system was evaluated as a safe and effective navigation tool for cup orientation. No information on clinical outcome or complications was provided.

In the next step, Ogawa et al. [31••] conducted a randomized controlled trial, where forty-six patients were randomly assigned to undergo acetabular cup placement during THA using either a marker-based AR navigation system or a conventional mechanical alignment guide. They found no differences in acetabular anteversion accuracy, and no clinically important differences in acetabular inclination.

### Trauma

The outcome of trauma surgery is highly dependent on exact anatomic reduction of the fractured bone fragments.

Ortega et al. [44] conducted a multicenter study including 50 patients using an HMD to display in-situ intraoperative fluoroscopic images acquired by a c-arm. With this technique, the surgeons’ attention left the operative field only five times compared to 207 times with conventional visualization. Radiation exposure was also significantly reduced.

Shen et al. [43] used an HMD for preoperative bending of osteosynthesis plates in 6 cases of pelvic fractures. The group reduced the fracture in a computer-assisted simulation and evaluated the optimal plate design. After determining the optimal plate shape, they bent the plate preoperatively by visualizing the optimal plate template with an HMD or monitor. After surgical sterilization, the pre-bent plates were used in the surgeries. For all patients, good anatomical reconstruction, good functional recovery, and no complications were reported. The surgery time was reduced by a mean of 10 min.

Von Heide [42] compared osteosynthesis, wiring, and implant removal surgeries using an AR application together with the camera-augmented c-arm [55] in 28 cases, in which registered imaging of the fractures was superimposed in-situ on a monitor and compared to 45 cases performed with conventional c-arm fluoroscopy. The group reduced radiation exposure by 46% (18 X-rays) using the AR visualization, but without observing any reduction in surgery time.

Weidert et al. [41] applied the camera-augmented c-arm for distal intramedullary nail locking in 42 bovine forelimb bones superimposing registered imaging of the X-rays in-situ on a monitor. Three surgeons with different levels of experience (beginner, intermediate, expert) conducted the experiments. The study group analyzed surgical accuracy, radiation dose, and surgery time. The main finding and benefit of their AR application was the significant reduction of radiation use, especially for novice surgeons.

### Orthopedic Oncology

Oncologic surgery is faced with a constant compromise between maintaining the safety margin required for sufficient tumor resection and excessive removal of functional tissue. Therefore, high surgical accuracy and exact execution are crucial for patient survival and optimal functional outcome.

Cho et al. [38] published an experimental study focusing on bone tumor resection. The group compared the feasibility of using AR with a tablet in comparison to a conventional tumor resection navigation method in 82 porcine cadaveric femurs. The conventional method consisted of an optical tracking system, a display, and a workstation. The group injected bone cement in the cadaver legs to simulate bone tumors. In the resections, the goal was to maintain a resection margin of 10 mm. The mean error was 1.71 mm in the AR group without any tumor violation and 2.64 mm in the conventional group with three tumor violations. The aimed oncologic margin of 10 mm was achieved in 90.2% of AR-guided resections and in 70.7% in the conventional group.

In addition to the cadaver evaluation, one clinical case of AR-navigated resection of a low-grade osteosarcoma in the diaphysis of the tibia was performed. The preoperative plan aimed for a 1.5-cm safety margin. Histologic workup showed a 1.4-cm margin proximally and a 1.7-cm margin distally.

The same research group described a further AR application for pelvic tumors [37]. As in their previous work, the resections were simulated by injecting bone cement in 18 porcine cadaver pelvises. The resection errors were classified into four grades: ≤ 3 mm, 3 to 6 mm, 6 to 9 mm, and > 9 mm or any tumor violation. After evaluation, average resection margin of the AR group was 1.59 ± 4.13 mm in comparison to 4.55 ± 9.7 mm in the control group. The group described the study as a proof of concept. Still, current results do not yet justify a clinical trial without further in-vivo animal studies.

Choi et al. [39] presented a similar study and resected 60 simulated bone tumors in porcine cadaver pelvises to compare AR-navigation using a tablet with conventional navigation. The conventional navigation method was not further described. As in the study of Cho et al., the aimed oncologic tumor resection margin was 10 mm. After analyzing the resected cadavers, AR showed a mean resection margin of 9.85 mm compared to a 7.11 mm resection margin in the control group.

## Conclusion

The technology of augmented reality is on the rise, and its application in orthopedic surgery has gained increasing attention opening up new opportunities in surgical planning and execution. The translation of pre-clinical results from proof of concept and feasibility studies to daily practice has been initiated. However, to this day the way AR and HMDs influence our concentration, perception and cognition is far from understood [71]. The sensory impression delivered through an HMD is a new experience to many surgeons. Avoiding overload of information and providing well-designed user interfaces will be necessary to smoothly integrate this technology in daily clinic practice. For final deployment and adoption, AR needs to be fully integrated into the surgical workflow [72].

The lack of robust and accurate registration and tracking processes which represent major limitations and refinements of this technology are needed to allow implementation of AR systems in the operating room. Here, inaccuracies can lead to misplaced virtual models making the navigation unreliable. Buggy navigation is widely known from other computer-assisted navigation approaches based on optical markers. Different new innovations are coming up; nevertheless, the technology itself needs further development in this direction. The ideal AR system should work automatically and allow not only surgical navigation but also error detection.
